# Pregnancy Outcomes in Women With Mechanical Valve Prostheses Using Vitamin K Antagonist Therapy: The Experience of the Salam Centre for Cardiac Surgery in Sudan

**DOI:** 10.3389/fped.2022.918547

**Published:** 2022-07-08

**Authors:** Nicoletta Erba, Sofia Gatti, Suha Abdelwahab Abdalla Hassan, Martin Langer, Liliane Chatenoud, Gina Portella, Raffaela Baiocchi

**Affiliations:** ^1^Emergency ONG ONLUS, Milan, Italy; ^2^Federazione Centri per la Diagnosi Della Trombosi e la Sorveglianza Delle Terapie Antitrombotiche (FCSA), Milan, Italy; ^3^Salam Centre for Cardiac Surgery, Khartoum, Sudan; ^4^Università Degli Studi, Milan, Italy; ^5^Laboratory of Clinical Epidemiology, Department of Public Health, Istituto di Ricerche Farmacologiche Mario Negri IRCCS, Milan, Italy

**Keywords:** anticoagulants-therapeutic use, pregnancy, mechanical heart valves, humanitarian medicine, Warfarin, maternal death, Salam Centre for Cardiac Surgery

## Abstract

Pregnancy and childbirth on anticoagulants after mechanical heart valve replacement present a high risk of complications for both mother and baby. On top of pregnancy worsening the mother's cardiac function, anticoagulant therapy itself is a crucial problem. A safe and effective anticoagulation regimen for both mother and fetus is not possible. The most effective drugs for preventing valve thrombosis are VKAs, whose dosage needs to be adjusted with frequent INR checks. Moreover, VKAs can have embryopathic and teratogenic action. Patients in follow-up and anticoagulant treatment at the Salam Centre for Cardiac Surgery in Sudan live spread out over a large area where transport to the Center is generally difficult; pregnancy treatment has, therefore, been adapted to the limitations of reality. Pregnancy is discouraged and contraception and therapeutic abortion are recommended, but this guidance frequently goes unheeded. Here we describe maternal and fetal outcomes in 307 consecutive pregnancies recorded by staff at the oral anticoagulant clinic (OAC) from April 2017 to November 2021. Out of 307 pregnancies, there were 15 maternal deaths (4.9%), 24 thrombotic events (7.8%) and 22 major bleedings (7.2%). Fifty pregnancies (16.3%) were terminated by therapeutic abortion. Only 47.6% of pregnancies had good maternal and neonatal outcomes. Data clearly show that, due to the complexity of pregnancy in women with mechanical heart valves and the scarcity of tertiary healthcare services in the area where patients live, maternal mortality is at an unacceptable level and requires a structured, multi-disciplinary intervention.

## Introduction

Mechanical heart valves (MHVs) replacing destroyed natural ones are life-saving for patients with advanced rheumatic heart disease (RHD). The downside of this surgical approach is the resultant need for life-long anticoagulant treatment, to limit the risk of thrombosis around the MHV, which can lead to severe valve dysfunction, embolism and stroke. Anticoagulant treatment has crucial intrinsic risks: if the dose is too low, the MHV function is put at risk of enhanced clotting; if the dose is too high, bleeding may occur spontaneously or after trauma. Both risks may be life-threatening, so careful management of anticoagulant therapy is essential. For long-term systemic anticoagulant treatment, Warfarin, a vitamin K antagonist (VKA) approved nearly 70 years ago, is still the drug of choice (1, 2). Warfarin is effective and cheap, but the treatment is complex and sometimes cumbersome. Patient compliance, frequent blood tests to check prothrombin time and international normalized ratio (INR), and dose adjustments are imperative (1, 2).

Moreover, VKAs are characterized by a significant, dose-dependent, placental drug transfer, (3) which can lead to embryo-/fetotoxicity and teratogenicity, (4) frequently leading to miscarriage, stillbirth and congenital malformations (Warfarin syndrome) (5–9). Intrauterine hemorrhage may also occur and lead to permanent damage. The balance between the need for anticoagulation to avoid or limit severe complications from MHV dysfunction and stroke for the mother, on the one hand, and a smooth pregnancy producing a healthy baby, on the other, is very fine. Some progress has been made in this field with the introduction of low molecular weight heparin (LMWH) in specific periods or throughout the pregnancy (1–3). This alternative anticoagulant treatment does not cross the placental barrier and is not dangerous for the child. However, it is reported to be less effective in preventing thrombosis in mothers, particularly where the dose cannot be adjusted weekly according to the level of factor anti-Xa activity (1, 2, 10, 11). Despite the limited data available from randomized controlled trials, the recent guidelines (1) recommend anticoagulation with LMWH and factor anti-Xa activity monitoring in the first trimester in patients requiring Warfarin doses of >5 mg/day; after the 36th week, a shift to LMWH is imperative regardless of the daily dose of warfarin. The unavailability of frequent anti-Xa activity monitoring and the high cost of LMWH in medium- and low-income countries (MLICs) make it challenging to follow these recommendations.

Our patients come from Sudan and neighboring states, most of which are low-income countries (LICs) with very limited healthcare systems that cannot support pregnant patients in need of anticoagulation. Moreover, many women live far from where they can get blood tests, counseling, drug prescription and drug supply and these difficulties greatly amplify the risk in pregnancies with anticoagulation compared to the same situation in high-income countries (10).

Women with MHVs treated with anticoagulants are at a high risk of death during pregnancy, at delivery and in puerperium; pregnancy is therefore strongly discouraged before surgery and at discharge after surgery. However, women are often eager to see their pregnancy through and, in some cases, are forced to do so due to social and family pressure.

Avoiding pregnancy as well as supporting pregnant women remain frequent and difficult tasks at the *Salam* Centre for Cardiac Surgery in Khartoum. This study aims to describe and analyze the outcome of pregnancies in women on anticoagulants in follow-up treatment at the *Salam* Centre and discuss possible improvements in the clinical path.

## Methods

The *Salam* Centre for Cardiac Surgery (12), a humanitarian project by the NGO EMERGENCY in collaboration with the Sudanese government, has been operating in Khartoum since 2007.

The Center offers surgical treatment free of charge for RHD and congenital heart disease to patients from Sudan and neighboring countries. Between the opening of the *Salam* Centre in 2007 and November 2021, 3,552 women underwent valve surgery and had one or two mechanical heart valves implanted, therefore requiring life-long anticoagulant treatment.

Patients scheduled for this type of surgery had pre-operative assessment and counseling for pregnancy (strongly discouraged) and contraception (strongly recommended). Counseling about contraception and pregnancy is repeated in the OAC at discharge. In case of pregnancy referred at the follow-up visit, therapeutic interruption is encouraged. In case of refusal, the following steps are implemented: full cardiac assessment is provided; Warfarin therapy is continued, with the addition of 100 mg of Aspirin per day (2): INR checks and dose adjustments are made every 2 weeks until the 36th week. At the 36th week, VKA is switched to full-dose LMWH. Patients have to rely on hospitals other than the *Salam* Centre for delivery and obstetric care. After delivery, VKA therapy is recommenced, and the patient is submitted for full cardiac assessment as soon as possible. However, patients often adhere only partly to this program, especially those living in regions far from the Center.

### Data Collection

In this retrospective study of routinely collected data, all consecutive pregnancies from April 2017 to November 2021 in patients with MHV implants and follow-up at the *Salam* Centre were included. The Center runs the OAC in collaboration with the Italian federation of anticoagulant clinics (FCSA). It provides all patients, free of charge, with counseling, management of anticoagulant treatment, INR checks and other necessary blood tests, VKA dose adjustment and rescue treatments in collaboration with cardiologists and cardiac surgeons (12).

Data on cardiac procedures, prescribed Warfarin doses, pregnancy outcomes, cardiologic follow-up, vital status and complications in patients during and after pregnancy [major (13, 14) and clinically relevant bleeding, thrombosis and embolism] were stored, together with the INR and warfarin prescriptions, in the dedicated software Parma^®^ (Werfen Italy).

Survival is reported as survival during pregnancy up to 6 weeks after delivery (according to the definition of maternal mortality); the outcome of “late maternal mortality” (maternal death between 6 weeks and 1 year post-partum) has been evaluated for the sub-group of patients with 1 year of follow-up after birth. Fetal/neonatal outcomes concern survival; no data about congenital malformations is available. Uncertainties regarding the outcome of babies are reported as unknown.

### Statistical Analysis

All data were entered and stored on Microsoft Excel files. Descriptive analyses are presented as frequencies, proportions, means and standard deviations, medians and interquartile ranges (IQR) where appropriate to characterize patients and their clinical outcomes. Proportions are compared using the χ2 test or Fisher Test, depending on variables distribution. For maternal outcomes occurred up to 6 weeks after delivery (i.e., major bleeding, thrombosis and maternal mortality), 95% confidence intervals (95% CI), are also presented. SAS 9.4 software (Inc., Cary, NC, USA) was used for all analysis.

## Ethics

The institutional, scientific ethics board of the University of Milan has approved this study and, due to the nature of retrospective chart reviews, waived the need for informed consent from individual patients.

## Results

Between April 2017 and November 2021, 307 pregnancies in 253 women were assessed at the OAC of the *Salam* Centre. All patients had mechanical heart valve prostheses: 15 isolated aortic valve prostheses (AVPs), 163 isolated mitral valve prostheses (MVPs) and 75 combined MVPs and AVPs. The average individual weekly dose of Warfarin was 43.1 ± 18.7 mg, while 40% of the women were prescribed a dose above 5 mg/day.

The median elapsed time from the operation to the first reported pregnancy is 4.5 years, ranging from one to 12 years.

Most women (206, 81.4%) had only one pregnancy, 47 (18.6%) patients had more than one pregnancy (40 had two, seven had three). Therefore, we analyzed the outcome of 253 pregnant women (mean age 28.1 years ± 6.6, range 14–50) and 307 pregnancies. There were no losses in the follow-up (FU) during pregnancy; nevertheless, 11 patients were lost to follow-up after delivery, namely 2 did not attend the 6 week-FU and 9 the 1 year-FU.

According to the definition of maternal death (up to 6 weeks after birth), the overall maternal survival was 95.1%; we reported 15 maternal deaths/307 pregnancies (4.9%), corresponding to 5.9 deaths/100 women. In the sub-group for which a follow-up 1 year after birth was available (234), we recorded nine further late maternal deaths (3.8%).

Maternal deaths, major bleeding and thrombosis are reported in Table 1, for all pregnancies and in strata of pregnancies outcome. Causes of death (mainly reported to us by relatives) are lacking in most cases: five valve thrombosis (three with documented diagnosis, two suspected), one intracranial bleeding, three infections and six unknown causes. Only major bleeding showed a significant different distribution according to pregnancy outcome, with a higher frequency after spontaneous or therapeutic abortion (12 and 12.9%, respectively), than after delivery (3.9%). On one occasion, it was responsible for maternal death. Thrombosis leading to death was more frequent than hemorrhage (33.3 vs. 6.7%). The therapeutic interruption was complicated by bleeding in 12% of the cases (Table 1); nevertheless, no significant association is found between the two events. Therapeutic interruption is related to thrombosis and blocked valves (*P* < 0.05) as most of the women (75%) were directed to perform an abortion once discovered the thrombosis. Only two patients had thrombosis after abortion as a proper bridging may have been omitted, or the intervention may have been performed outside hospitals.

**Table 1:** Maternal outcome in pregnant women with MHV: overall and in strata of pregnancies outcome.

	Maternal death^*^	**Major bleeding ^¥^**	**Thrombosis^†^**
	***N***	***N***	***N***
	**(%; 95% CI)**	**(%; 95% CI)**	**(%; 95% CI)**
All pregnancies (*n* = 307)	15 *(4.9)* *(4.9; 2.8–7.9)*	22 *(7.2)* *(4.5–10.7)*	24 *(7.8)* *(5.1–11.4)*
Therapeutic abortion (*n* = 50)	3 *(6.0)* *(1.3–16.6)*	6 *(12.0)* *(4.5–24.3)*	8 *(16.0)* *(7.2–29.1)*
Miscarriage/Fetal demise <28 weeks (*n* = 70)	2 *(2.9)* *(0.4–9.9)*	9 *(12.9)* *(6.1–23.0)*	4 *(5.7)* *(1.6–14.0)*
Delivery >28 weeks/Post-partum (*n* = 180)	3 *(1.7)* *(0.4–4.8)*	7 *(3.9)* *(1.6–7.9)*	12 *(6.7)* *(3.5–11.4)*
*P-values* ^§^	*0. 196*	*0.019*	*0.090*

* *Seven women died during pregnancy*.

¥ *17 metrorrhagia, one hemorrhagic stroke, four other causes requiring blood transfusion*.

† *23 valve thrombosis, one ischemic stroke*.

§ *Fisher exact Test for maternal death and thrombosis, Chi-Square for major bleeding*.

TTR calculated with the Rosendaal method shows a median TTR of 28% (13–46) during pregnancy, a value considered particularly low, especially when compared to the TTR before pregnancy (44%, 25–59). Moreover, a significant association was found between TTR > 46% (IV Quartile) and a reduced incidence of adverse events such as thrombosis and bleeding (*P*-value 0.0128).

A limited number (153/307, 49.8%) of pregnancies resulted in babies born alive (Table 2).

**Table 2:** Fetal outcome.

	**Overall**	**One pregnancy**	**Two or three pregnancies**
	***N* (%)**	***N* (%)**	***N* (%)**
All pregnancies	307	206	101
Born alive	153 *(49.8)*	112 *(54.4)*	41 *(40.6)*
Therapeutic abortion (TA)	50 *(16.3)*	30 *(14.6)*	20 *(19.8)*
All pregnancies–excluding TA	257	176	81
Born alive	153 *(59.5)*	112 *(63.6)*	41 *(50.6)*
Miscarriage/Fetal demise <28 weeks	70 *(27.2)*	39 *(22.2)*	31 *(38.3)*
Stillbirth/neonatal death	24 *(9.3)*	16 *(9.1)*	8 *(9.9)*
Maternal death during pregnancy	7 *(2.7)*	7 *(4.0)*	0 *(0)*
Unknown	3 *(1.2)*	3 *(1.7)*	0 *(0)*

Only 133 out of the 236 (56.4%) women, who survived pregnancy and the first 6 weeks post-partum, gave birth to at least one alive baby (119 to one, 13 to two, and one to three babies); 104 mothers failed to do so, while three babies born alive lost their mothers at birth.

As for termination of pregnancy on the recommendation of cardiologists, it was agreed to by 37 patients (28.5%) counseled in the first trimester of each pregnancy and by 8 (15.4%) in the second trimester (OR 2.18, CI 0.95–5.37 *P*-value = 0.09).

## Discussion

To become a mother is a natural desire all over the world. In many medium- and low-income countries, and indeed high-income ones, the importance placed on women having children, and the social pressure on them to do so, is very strong. In Sudan and neighboring countries, having children, often many children, is a sign of prestige for women; not having children is often seen as indicative of a lack of values by women's families and society, and can impact very heavily on their self-esteem and quality of life. Moreover, in LICs, having a lot of children can be a substitute for the weak support provided by the national welfare system to elderly and fragile family members.

Most women taking anticoagulants after heart surgery and mechanical valve implants have to live with this reality. Many of them are very young and have had difficult lives even before surgery. Streptococcal infection, rheumatic fever and RHD are mostly found among the poorest, and illness hinders schooling and acquisition of skills. Heart surgery and mechanical valves save lives, but they make life-long treatment a necessity and any return to normality only partial, with a higher risk of complication-related morbidity and mortality.

During the observation period, 253 women who had been referred to the OAC for VKA management due to MHVs became pregnant despite repeated advice to avoid doing so. When prevention fails, therapeutic interruption is recommended to pregnant women (and frequently refused). A significantly higher adherence to recommendations (even if not statistically significant) has been noticed in patients who received counseling in the first trimester of pregnancy; they had therapeutic abortions in almost three times as many cases as women counseled in the second trimester. This can be due to factors related to both women's attitude (intentional delay in informing the cardiologist, in order to preserve their pregnancy; increased confidence in a smooth pregnancy as the months go by) and significant difficulties finding doctors/facilities willing to interrupt pregnancies in the second trimester of pregnancy. Pregnant women refusing abortion have a complete cardiological visit; administration of VKA is continued as the most effective and safest anticoagulation treatment for the mother, especially as weekly anti-Xa activity monitoring and dose adjustment are not possible (1). The prescribed dose of Warfarin is in 40% of the ladies higher than the threshold of 5 mg/day and the risk of fetal damage (15, 16) is expected to be more frequent. However, our limited data, with many values missing, do not allow conclusions on this point.

Nearly half (154/307, 50.2%) of all pregnancies are unsuccessful. Only 133/236 (56.4%) mothers gave birth to at least one live baby. Nearly one out of five mothers (19.8%) repeatedly tried to have a child, but only 41 children were born alive after their collective 101 pregnancies. The price paid by these mothers is very high in terms of suffering, frustration and survival (Table 1): we registered 15 maternal deaths/307 pregnancies (4.9%).

The most frequent and life-threatening complications were valve thrombosis, which might have been caused by low compliance to oral anticoagulant treatment (OAT) once the patients became aware they were pregnant. Indeed, information on the embryo-/fetotoxicity of VKAs can lead to patients giving up the medication prescribed for them or taking it irregularly. Haemorrhagic complications occurred mostly around delivery or miscarriage/therapeutic abortion. Haemorrhagic complications had lower mortality strictly speaking, but sometimes led shortly after to thrombosis because of improper anticoagulation management after bleeding in hospitals, where medical staff are not trained to find the balance between the risk of bleeding and thrombosis.

In fact, rather than a potential improvement from complicated and costly anticoagulation strategies with probable but uncertain benefits to both mothers and babies, (17) the lack of competent clinical support from tertiary care centers (1, 2) is the most pressing of our patients' needs. Maternal mortality (4.9%) is much higher than in the ROPAC cohort (18) (1.4% of 212 patients, most of them from M-LICs, but not from sub-Saharan Africa). However, a comparison has limited value as their observation period is much shorter (follow-up until 1 week after delivery); pregnancies, however, ending with a baby being born alive were only 58%, not too far from our 50.3% (59.5% considering only pregnancies without women who had therapeutic abortion).

In the ROPAC (18) cohort, over 40% of pregnant women experienced severe complications, but most were treated and survived. Higher, but still far from our mortality rate, are the data from the metanalysis by Lawley et al. (19) on 256 patients with MHVs; they report a maternal mortality of 1.8 (CI 0.5–3.7) deaths/100 pregnancies. Once again, comparison with our data is limited, as no details about length of post-partum follow-up are reported. We register 4.9 deaths/100 pregnancies, which is above the upper limit of the confidence interval.

It emerges very clearly that we have to improve maternal survival before all else. Safeguarding mothers' lives must become our principal commitment. Where long-term anticoagulation cannot be avoided, and contraception is not accepted, a nationwide obstetric support network seems the only solution; the *Salam* Centre can advise hospitals on anticoagulation management in case of hemorrhage or thrombosis provided there is a mutual relationship of trust. A similar network is in place for cardiology but is needed also for obstetrics.

More efforts could be made to improve women's awareness about health issues related to pregnancy. Standardized counseling, focusing on self-engagement, can strengthen relationships between patients and caregivers, improve adherence to therapy and contraceptive indications, and ensure timely communication about pregnant status.

## Strengths and Limitations of the Study

We report on a very large, single-center experience of treatment for mothers taking anticoagulants after heart-valve surgery in sub-Saharan Africa. The analysis and the findings may help point the way to effective solutions for improving outcomes.

On the other hand, it is clear that there are many limitations, primarily due to the retrospective nature of the study and the difficulties in obtaining information from other hospitals or patients/relatives about patients' outcomes. This is particularly evident when it comes to causes of death and assessment of child morbidity.

## Data Availability Statement

The original contributions presented in the study are included in the article/supplementary materials, further inquiries can be directed to the corresponding author/s.

## Ethics Statement

The studies involving human participants were reviewed and approved by University of Milan. Written informed consent for participation was not provided by the participants' legal guardians/next of kin because: The institutional ethics board of the University of Milan due to the nature of retrospective chart reviews, waived the need for informed consent from individual patients.

## Author Contributions

NE organized and supervised the Oral Anticoagulant Clinic, contributed to the study concept, data collection, interpretation, writing, and supervision. SG contributed to the data cleaning, managed the database, contributed to interpretation, and contributed language revision. SH contributed to data collection and interpretation. ML contributed to the study concept, writing, and supervision. LC contributed to the statistical analysis. GP contributed to the study design and supervision. RB contributed to the design, elaboration of the data, writing, and supervision. All authors contributed to the article and approved the submitted version.

## Conflict of Interest

The authors declare that the research was conducted in the absence of any commercial or financial relationships that could be construed as a potential conflict of interest.

## Publisher's Note

All claims expressed in this article are solely those of the authors and do not necessarily represent those of their affiliated organizations, or those of the publisher, the editors and the reviewers. Any product that may be evaluated in this article, or claim that may be made by its manufacturer, is not guaranteed or endorsed by the publisher.
